# Highly improved electrocatalytic oxidation of dimethylamine borane on silver nanoparticles modified polymer composite electrode

**DOI:** 10.3906/kim-1906-23

**Published:** 2020-02-11

**Authors:** Çağrı Ceylan KOÇAK, KOÇAK Süleyman, Şükriye KARABİBEROĞLU, Zekerya DURSUN

**Affiliations:** 1 Bergama Vocational School, Dokuz Eylül University, İzmir Turkey; 2 Department of Chemistry, Faculty of Science and Letters, Manisa Celal Bayar University, Manisa Turkey; 3 Department of Chemistry, Faculty of Science, Ege University, İzmir Turkey

**Keywords:** Dimethylamine borane, silver nanoparticles, poly(aminophenol), fuel cell, scanning electron microscopy

## Abstract

Dimethylamine borane (DMAB) is a promising fuel alternative for fuel cell applications. In this work cyclic voltammetric behavior of DMAB was investigated on the polymerized aminophenol film decorated with Ag nanoparticles in alkaline media. The polymer film was formed on the glassy carbon electrode by electrochemical technique and then, the surface was modified with Ag nanoparticles. The surface of the modified electrode was identified by scanning electron microscopy, transmission electron microscopy, X-ray photoelectron spectroscopy, and electrochemical impedance spectroscopy techniques. The developed electrode has displayed high electrocatalytic activity for DMAB oxidation in alkaline media depending on the supporting electrolyte concentration. Experimental parameters such as cycle number used in electropolymerization of p-aminophenol, deposition of Ag nanoparticles and supporting electrolyte were optimized.

## 1. Introduction

Fossil-based energy resources give rise to environmental problems that led researchers to find alternative renewable energy sources. In recent years, fuel cell developments have received great attention due to their low emissions to environment and high efficiencies. Fuel cells convert chemical energy stored in fuels to electrical energy and therefore require a constant source of fuel to sustain the chemical reaction [1–3].

Dimethylamine borane (DMAB) has amine odor and a chemical formula of (CH3)2 NH:BH3 [4]. DMAB is used in wide range of applications as a reductant [5–7] and is also used as a fuel in fuel cell applications [8,9]. In recent years, studies on the use and research of boron-derived fuels have become widespread all over the world [10–12]. DMAB is a boron derivative fuel and it is important to examine its possible behavior as an alternative energy source. For this purpose, some researchers have studied the oxidation reaction of DMAB [9,13,14]. Generally, from the reaction of DMAB in the alkaline solution, the hydroxytrihydroborate intermediate anion occurs [9,14,15]. Two mechanisms have been proposed for DMAB oxidation on the different gold electrode. Plana and Dryfe [9] and Plana et al. [14] reported that the hydroxytrihydroborate anion is either converted to water with 6 electron yields, or a lower-efficiency oxidation process may produce molecular hydrogen with 3 electron yields. The general mechanism of DMAB has been proposed as an integer between 3 and 6 [9,14]. The anode reaction of the direct DMAB fuel cell is the direct oxidation of DMAB in alkaline medium as follows:

(1)(CH3)2NHBH3(aq)+OH(aq)-→BH3OH(aq)-+(CH3)2NH(aq)

The hydroxytrihydroborate anion generally follows the possible two ways: First one is six-electron pathway (high efficiency) [13,16,17]:

(2)BH3OH(aq)-+6OH(aq)-→B(OH)4(aq)-+3H2O(l)+6e-

The second pathway yields three electrons and molecular hydrogen (lower efficiency):

(3)BH3OH(aq)-+3OH(aq)-→B(OH)4(aq)-+(3/2)H2(g)+3e-

The hydrolysis of DMAB resulted in the formation of hydrogen gas as the anode fuel (Eq. (3)). On the other hand, direct DMAB oxidation provides an improved cell voltage compared with oxidizing molecular hydrogen. Competing chemical hydrolysis of DMAB with evolution of hydrogen, given by Eq. 3, is expected to be minimal at pH values in excess of 12.

To date, there have been only a few studies which attempt to explain the mechanism of DMAB direct electrooxidation. In one of these studies, the oxidation of DMAB on polycrystalline gold electrodes was investigated by Plana and Dryfe [9]. Two sequential oxidation processes took place on gold surface. Three e− are removed from DMAB, so H2 is not oxidized at low potentials. A six-electron was extracted from molecule at high potential. Another study about direct electrooxidation of DMAB was conducted by Nagle and Rohan [13]. It explained that an overall coulomb number of six has been determined from the data acquired using the diffusion coefficient determined and the number of electrons in the oxidation reaction depends on the OH–DMAB ratio and the hydrogen content becomes increasingly important as this ratio decreases. The direct electrooxidation of DMAB was also investigated with Au and Pt bulk surface by Finkelstein et al. [18]. This study assumed that DMAB completely dissociates as in Eq. (1). Another finding is that DMAB oxidation has two distinct oxidation processes taking place at low potential region and high potential region.

Metal nanoparticles have attracted great attention for their unique physical and chemical properties owing to their high surface area to volume ratio that differs from their bulk forms [19,20]. Wide range of techniques such as microemulsion [21], sol-gel [22], thermal reduction [23], metal vapor [24], and chemical reduction [25,26] have been used for their synthesis. Electrochemical synthesis, on the other hand, has drawn particular attention as an inexpensive and rapid technique with an advantage of controlling over the particle size by changing the conditions. Metal nanoparticles are usually deposited on a support for stability such as conducting polymers [27]. Pt, Au, Pd, etc. are widely studied metal nanoparticles in various kinds of applications [28–31]. Although the efficiency obtained in the presence of these metals is high, the cost considerably increases. Therefore, there is a need for cheaper and highly effective alternative catalytic surfaces. Ag has an important place among other alternative metals due to its good catalytic activity, biocompatibility, ability for accelerating the electron transfer and electro-conductibility [32]. Different silver forms offered good catalytic activity for such analytes [33–35]. In addition, the price is quite low compared to precious metals, which is an important parameter for applications [36]. In this regard, Ag nanoparticle modified surface is designed as a candidate for an active surface to energy application.

Polymers are promising materials for the preparation of nanocomposites. Conducting polymers have attracted significant attention because of their chemical, mechanical, optical, and electrical properties [37–39]. Due to their unique properties, a variety of conducting polymers are used in analytical applications [40] as well as catalytic surfaces in fuel cells [41–44]. Recently, some reports have been published by our research group about application of polymer modified electrodes on neurotransmitter detection [45], oxygen reduction [46], ammonia borane investigation [47], and antimony detection [48]. In each substrate polymeric structure has provided long-term stability for metal nanoparticles. Compared to all other polymers, electroactive aminophenol polymer (PAP) is remarkable in its various properties. In different environments, it has the ability to form highly conductive film on different surfaces [49–51]. Active functional groups in its structure both facilitate the polymerization on the electrode surface and form a selective surface for many analytes [46,52–54]. PAPmodified surfaces maintain their conducting properties for a long time as compared to many conductive polymers [55]. Chemically stable homogeneous PAP films with controlled thickness can be achieved with electrochemical modification [56]. When all these mentioned features are evaluated, PAP stands out as a preferred modifier.

Present work describes the fabrication of oxidized Poly (p-aminophenol) film modified electrode decorated with Ag nanoparticles (AgNPs) for the investigation of DMAB oxidation in alkaline solutions. The electrode surface was characterized with scanning electron microscopy (SEM), transmission electron microscopy (TEM) energy-dispersive X-ray spectroscopy (EDX), X-Ray photoelectron spectroscopy (XPS) and electrochemical impedance spectroscopy (EIS). Experimental parameters both in electropolymerization and DMAB oxidation were optimized and the results of electrocatalytic oxidation of DMAB at the AgNPs-modified oxidized polymer film electrode were compared with those obtained with bare GCE and polymer film electrode.

## 2. Materials and methods

### 2.1. Reagents and instrumentation

The monomer, p-aminophenol, was obtained from Fluka and the fuel, DMAB, was purchased from Alfa Aesar. NaOH reagent was obtained from Raidel De Haen. Sodium dodecylsulfate (SDS) and HClO _4_ reagents were of analytical-reagent grade and supplied from Sigma. AgNO _3_ was purchased from Carlo Erba. All the solutions were prepared using ultrapure water. All electrochemical experiments were carried out under nitrogen gas.

Autolab 302N was used as voltammetric analyzer for measurements. Three electrode systems consisting of a working electrode, a platinum wire counter electrode, and an Ag/AgCl (sat. KCl) reference electrode were employed in measurements. Glassy carbon electrode (0.0707 cm ^2^ ) was obtained from BASi. The surface characterization was examined by using Thermo K-Alpha-Monochromated high-performance XPS spectrometer (XPS), JEOL JEM-ARM200F transmission electron microscopy (TEM), and Philips XL30 SFEG scanning electron microscopy (SEM).

### 2.2. Electrochemical measurements

Cyclic voltammetry (CV) and chronoamperometry were used for electrochemical measurements. CVs were carried out between –1300 mV and 0 mV with 50 mV s ^-1^ scan rate in 2.0 M NaOH containing DMAB. Chronoamperometric responses were recorded in a 2.0 M NaOH solution at peak potentials of Ag wire and AgNPs/PAP/GCE towards DMAB with 0.5 s time interval.

### 2.3. Preparation of working electrodes

Before modification, the GCE was polished with alumina slurry (0.05–3 μm) and rinsed with pure water. For removing any residues, the electrode was subjected to ultrasonication in ethanol (1:1, v/v) and ultrapure water. The GCE was then immersed in a 5 mM SDS and p-aminophenol containing 0.5 M HClO _4_ solution and electrochemical deposition of poly(p-aminophenol) on GCE was performed by cyclic voltammetry technique as given elsewhere [57]. Polymerized film was obtained by consecutive 35 potential cycles with 100 mV s ^-1^ scan rate from –0.5 V to 2 V. The resulting electrode was abbreviated as PAP/GCE.

AgNPs were deposited on the PAP/GCE surface using electrochemical reduction of silver ions from 2 mM AgNO _3_ on the PAP/GCE in 0.1 M HNO3 solution. It was performed by ten repetitive cyclic voltammograms in the potential range of 0.3 V and –0.9 V.

## 3. Results and discussion

### 3.1. Surface characterizations of electrodes

**Figure 1:**
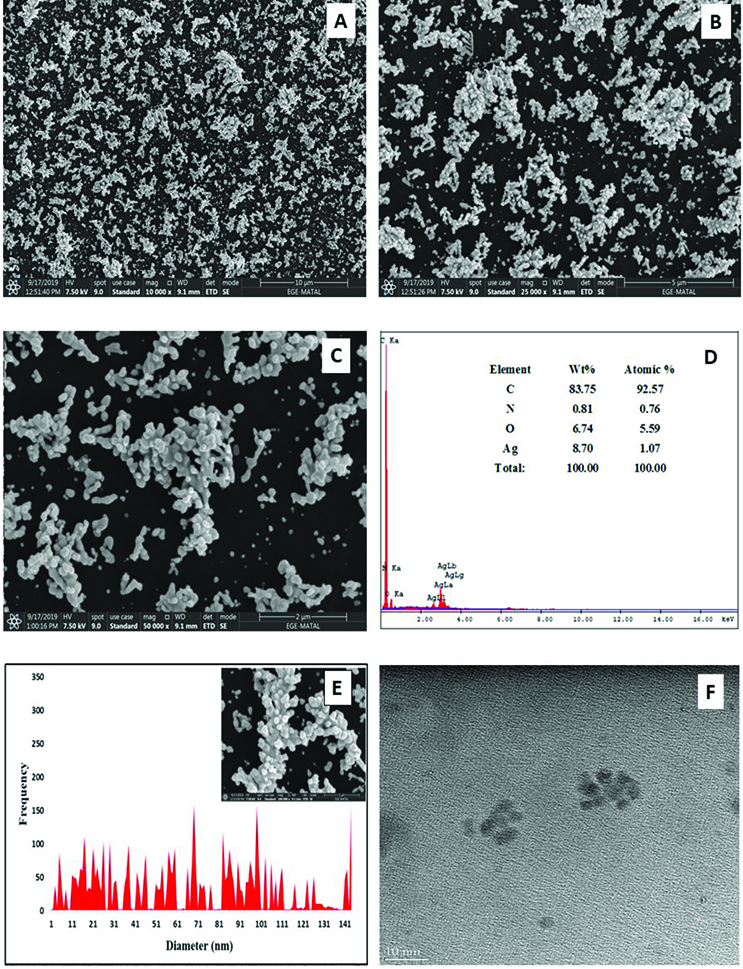
SEM images with different magnifications (A-C), EDX results (D), size distribution (shaded bars) of AgNPs on the PAP surface and TEM image (F) of AgNPs/PAP/GCE.

The surface morphologies of the electrodes were characterized by XPS, SEM, TEM, and EIS. Figures 1A–1C show the SEM images of AgNPs/PAP/GCE with different magnifications which exhibit the good surface coverage with polymer film and AgNPs. Spherical AgNPs are distributed homogeneously on the polymerized electrode surface. The presence of Ag element on the PAP/GCE surface was analyzed by Energy Dispersive X-ray (EDX) spectroscopy (Figure 1D). The weight gain of the PAP/GCE because of the Ag loading was found nearly 8.7%. The overall data has confirmed that uniform coverage of Ag on the PAP/GCE can be achieved with cyclic voltammetry. The size distribution of the Ag nanoparticles obtained from SEM analysis was shown in Figure 1E. The particle size ranges from 2 to 160 nm and the average particle size was found approximately 100 nm. Moreover, TEM image of AgNP-modified PAP surface (Figure 1F) exhibited small spherical and flower-like structures related to the AgNPs. Comparison of SEM and TEM images point out that the Ag metal particles’ diameters were not identical because of the application of different preparation procedures for SEM and TEM samples. For the SEM measurements, the electrochemically prepared composite surface was scanned by the SEM system; thus, the AgNPs/PAP/GCE surface was maintained. In addition, in TEM measurements, the electrochemically prepared polymer and Ag metal particles’ content were transferred to ethanol solvent by scraped from the electrode surface with a sharp knife and then TEM grids. The Ag metal particles should be liberated to smaller particles. Accordingly, number of the Ag metal particles in the SEM image was higher than that in the TEM image.

**Figure 2:**
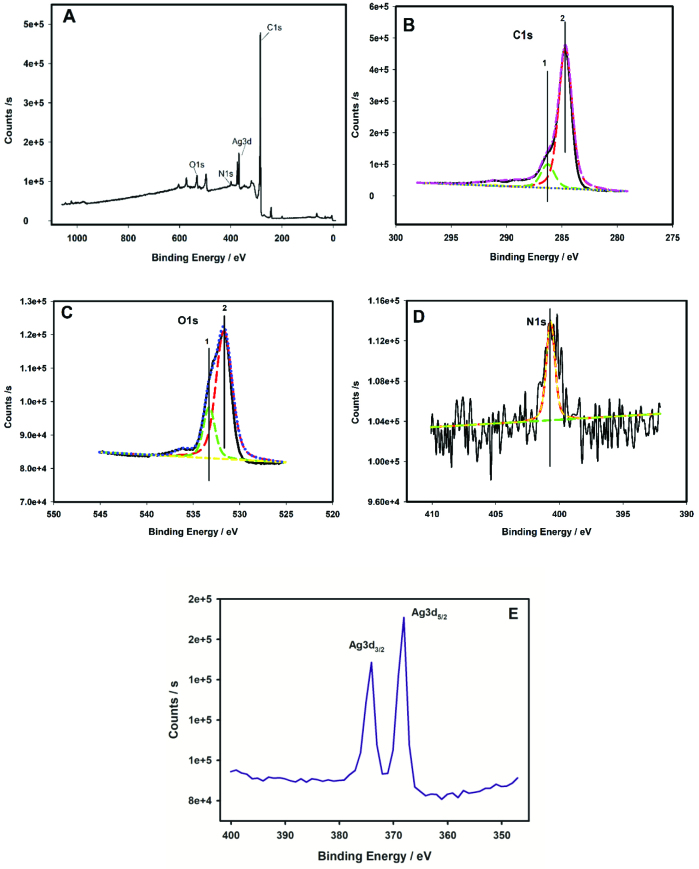
(A) XPS survey spectrum of AgNPs/PAP/GCE; core level spectra of (B) C1s, (C) O1s, (D) N1s, and (E) Ag3d.

XPS technique provides important information about bonding characteristics of the film and oxidation states of Ag nanoparticles. Figure 2A shows the wide spectrum of poly(p-aminophenol) film. It is evident that Ag3d, C1s, O1s, and N1s signals are present in polymer structure. For each curve-fitted region, the component peaks are numbered and chemical state assignment was identified based on literature data [58–60]. The curve fitting of the C1s peaks for PAP/GCE has been done by assuming two components at 284.7 and 286.2 respectively (Figure 2B). These values could correspond to C-N and C-O groups, on the basis of the PAP chemical formula. The fitted O1s spectra of the PAP modified GC electrodes were shown in Figure 2C. Two oxygen species were observed with the PAP/GCE (Figure 2B-1) at BE 531.7 and 533.3 eV. The lower BE signal (531.7 eV) could be assigned to –C-O bond. The BE at 533.3 eV could be due to typical of phenolic/ether oxygen. Figure 2D shows the fitted N1s spectra indicating one component centered at 400.2 eV, a binding energy typical of C-N groups in the polymeric chain. Figure 2E shows the XPS spectra of AgNPs/PAP/GCE in Ag3d region. Ag doublets of Ag3d _5/2_ and Ag3d _3/2_ were observed at binding energies of 368.39 and 374.08 eV, respectively. The results verified that Ag species inside the aminophenol polymer film were chiefly dispersed as metallic Ag [61,62]. Moreover, the amount of Ag was found as 34.4 μg cm ^-2^ according to the experimental results.

**Figure 3:**
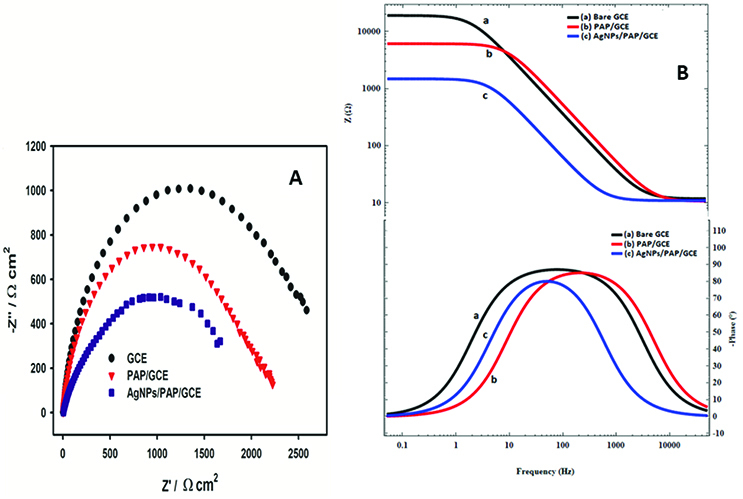
A) Normalized Nyquist plots B) Bode plots of bare and modified electrodes in 2.0 M NaOH containing 5.0 mM DMAB (frequencies: from 50 000 to 0.05 Hz).

EIS was performed in 5 mM DMAB containing 2 M NaOH solution in the frequency range of 50,000 to 0.05 Hz. The normalized Nyquist plots were represented in Figure 3A and results were fitted with (R(RC)) equivalent circuit. The highest electron transfer resistance was obtained at bare GCE. This value was decreased with polymer coating on the surface and reached its smallest value with subsequent Ag modification. As seen from the results, lower solution resistance and DMAB oxidation impedance indicate that DMAB is more easily oxidized on the AgNPs/PAP/GCE surface as compared to the GCE and PAP/GCE. In addition, Bode plots were shown in Figure 3B. They reveal the signs of redox activity both in the modulus of the impedance and the phase angle. The double sigmoid is visible at low frequency in modulus impedance. According to the phase angle, capacitive behavior is observed over a narrow frequency range and where a second capacitive contribution is seen between 1 and 10 Hz. This behavior is indicative of residual, albeit hindered, redox activity at the electrode surface. The impedance value was increased with the modification of PAP and AgNPs on the GCE surface. This behavior was found related with the faster electron transfer rate. At the intermediate frequencies the phase angle value remains almost the same for all electrodes. The same phase angle indicates that all electrodes have the same capacitive properties.

### 3.2. Electroactive surface area

In this study, the electroactive surface area (ESA) was via CV by employing the increasing scan rates on Ag- NPs/PAP/GCE in 0.1 M KCl containing 0.5 mM K _3_ Fe(CN) _6_ /K _4_ Fe(CN) _6_ couple. K _3_ Fe(CN) _6_ /K _4_ Fe(CN) _6_ involves one electron per molecule redox reaction [63]. Based on the obtained results, square root of scan rate versus peak current plot was drawn. ESA of the bare and modified electrodes were calculated with the help of Randles–Sevcik equation (4):

(4)ip=(2.69105n3/2ACD1/2v1/2

(diffusion coefficient) was taken as 6.70 ×10 ^-6^ cm ^2^ s ^-1^ , K _3_ Fe(CN) _6_ /K _4_ Fe(CN) _6_ concentration (C) was added to the equation, n is the transferred electron number in the redox system was taken as 1, active area (A) is the only unknown component in the equation that calculated from the result of the equation [64]. ESA of the electrodes were found as 0.056 cm ^2^ , 0.290 cm ^2^ , and 0.574 cm ^2^ for GCE, PAP/GCE and AgNPs/PAP/GCE, respectively. Presence of PAP and AgNPs lead to an increase on the electroactive area of GCE. The larger electroactive surface area confirms the higher activity toward DMAB oxidation.

### 3.3. Electrocatalytic oxidation of DMAB

**Figure 4:**
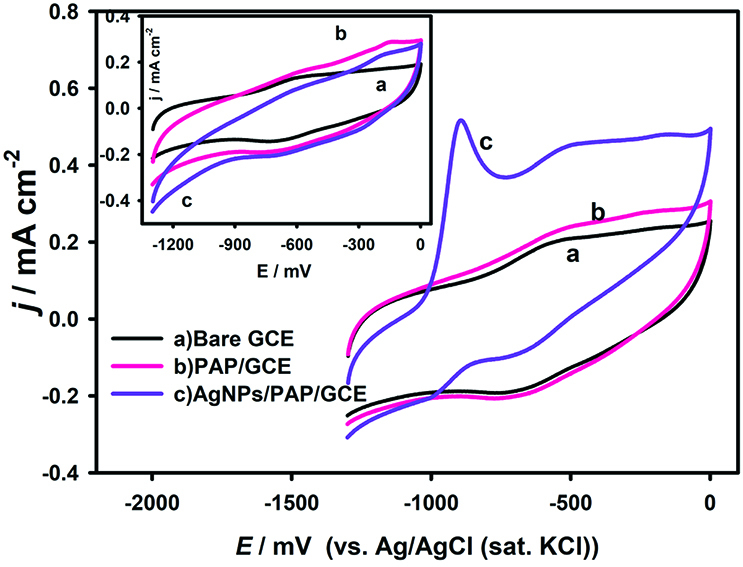
CVs of bare GCE, PAP/GCE and AgNPs/PAP/GCE in 2.0 M NaOH containing 1.0 mM DMAB. Inset: Background voltammograms.

The performance of the electrodes towards DMAB oxidation was tested by cyclic voltammetry in alkaline media. Figure 4 demonstrates the cyclic voltammograms obtained with the bare, PAP/GCE, and AgNPs/PAP/GCE electrodes in the presence (Figure 4) and absence (Figure 4-inset) of 1 mM DMAB solution in 2 M NaOH. The AgNPs/PAP/GCE has displayed an excellent catalytic activity towards DMAB oxidation in comparison to the bare electrode and PAP/GCE. A well-defined irreversible oxidation peak was observed at –916 mV with 0.36 mA cm ^-2^ current value at the AgNPs/PAP/GCE, while there is no response obtained at bare and PAP/GCE for DMAB oxidation.

**Figure 5:**
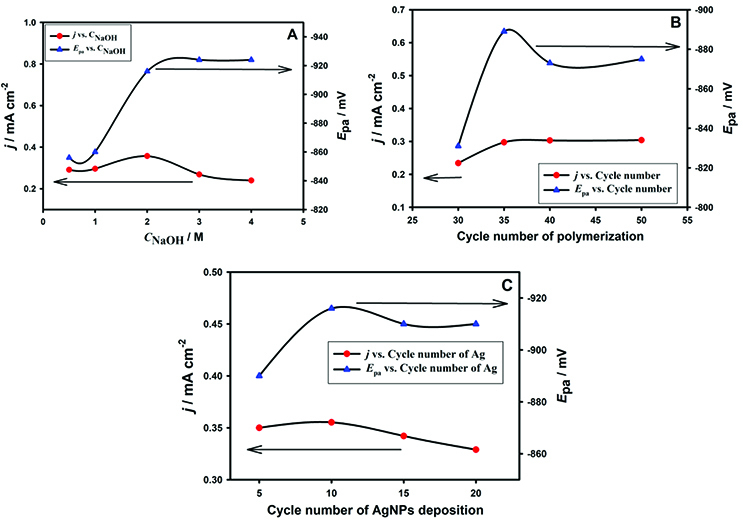
Anodik peak potential and current values of DMAB vs. A) NaOH concentration, B) Cycle number of PAP polymerization, and D) Cycle number of Ag deposition (AgNPs/PAP/GCE in 2.0 M NaOH containing 1 mM DMAB with 50 mV s ^-1^ ) .

In fuel cell studies, alkaline media is preferred for operational purposes. The effect of NaOH concentrations on the DMAB oxidation was investigated in the concentration range of 0.05 to 4.00 M (Figure 5A). The peak currents related with the DMAB oxidation have shown an increase up to 2 M and then decreased for more concentrated solutions of NaOH. The change in the peak current was accompanied by the shift in the peak potential by increasing NaOH concentration. It has been known that DMAB is relatively stable at high pH values [9]. Therefore, 2 M NaOH medium was selected as the optimum supporting electrolyte since it provides high peak current and a satisfactory shift in the peak potential. Electrochemical polymerization offers an advantage of tuning the polymeric film thickness and nanoparticle size/distribution which are important in terms of catalytic activity. The surface coverages of PAP/GC electrodes were adjusted by changing the cycle numbers used in polymerization from 30 to 60 in the studied potential range. Figure 5B demonstrates the effect of polymerization cycle number on the oxidation peak of 1 mM DMAB in 2 M NaOH. As can be seen clearly in Figure 5B, the peak current obtained for DMAB has given a plateau between 35 to 50 cycles while the desirable shift in peak potential was observed at 35 cycles. Therefore, the potential was cycled from –0.5 to 2 V for 35 consecutive scans with a rate of 100 mV s ^-1^ in further modification studies.

Another parameter to be optimized is the cycle number of Ag deposition which is used for nanoparticle formation. The cycle number varied from 5 to 20 and resulting peak characteristics were plotted in Figure 5C. DMAB oxidation peak current reached its maximum value with the satisfying potential value with 10 cycles of Ag deposition on PAP/GCE.

**Figure 6:**
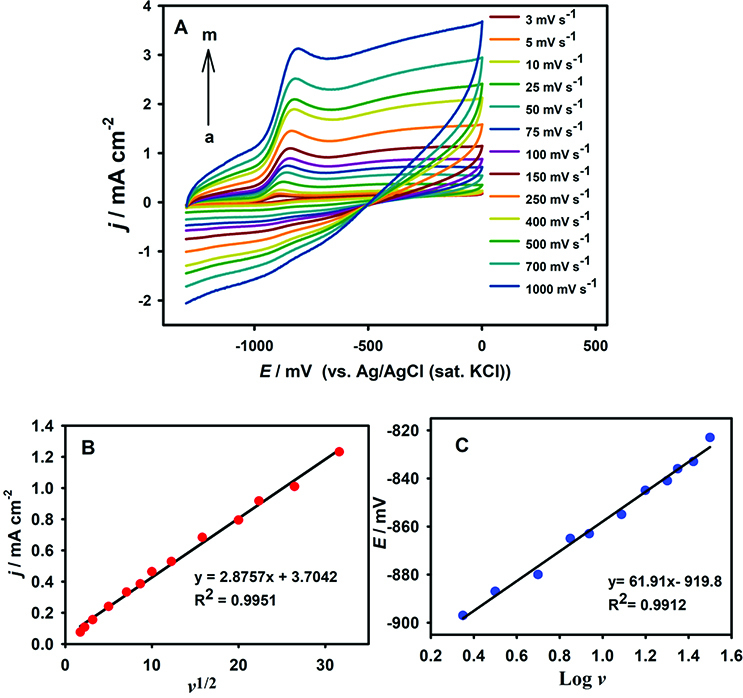
A) CVs of 1.0 mM DMAB oxidation in 2.0 M NaOH at increasing scan rates. a-m: 3–1000 mV s ^-1^ on AgNPs/PAP/GCE. Plots of the B) Electrocatalytic current of AgNPs/PAP/GCE vs. the square root of scan rate (v1/2) , and C) Oxidation potential vs. Log v.

Cyclic voltammetric response of AgNPs/PAP/GCE in 1 mM DMAB containing 2 M NaOH with various scan rates were shown in Figure 6A. The peak current of DMAB was directly proportional to the square root of scan rate in the range of 3–1000 mV s ^-1^ . These data show that the electrode reaction was controlled by diffusion of DMAB to electrode surface (Figure 6B). The plot of peak potential vs. logarithm of scan rate was also shown in Figure 6C. The logarithm of the scan rate has a linear dependence with peak potential; therefore, the results showed that an irreversible electrode reaction occured on the AgNPs/PAP/GCE.

**Figure 7:**
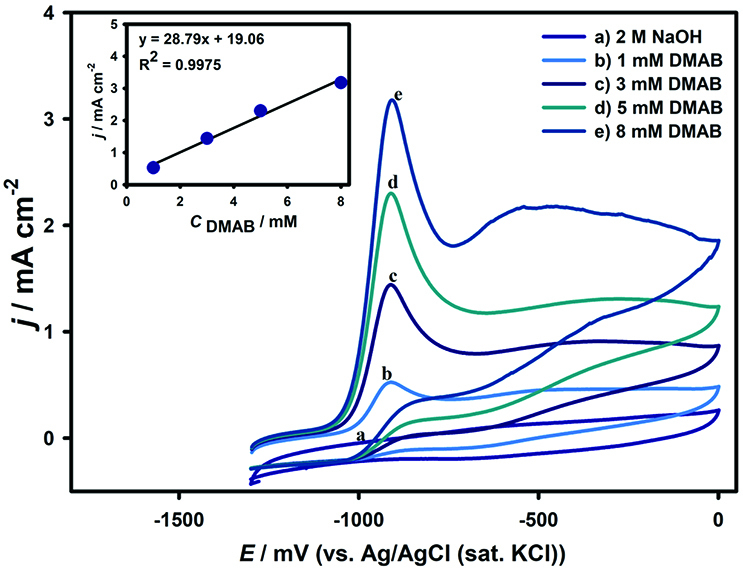
Cyclic voltammograms of a) Background response in 2.0 M NaOH, b) 1.0 mM, c) 3.0 mM, d) 5.0 mM, and e) 8.0 mM DMAB oxidation at AgNPs/PAP/GCE. Inset: plot for the peak current of AgNPs/PAP/GCE versus the concentration of DMAB.

The voltammetric response and corresponding calibration plot of peak currents vs. concentrations are shown in Figure 7. The curves b–e corresponds to the DMAB concentration of 1.0, 3.0, 5.0, and 8.0 mM, respectively where curve a displays the background response of electrode. The oxidation peak currents increase linearly from 0.36 to 2.33 mA cm ^-2^ with a correlation of 0.9975 by changing DMAB concentration in a range of 1–8 mM. The overall data indicates the efficiency of AgNPs/PAP/GCE electrode for DMAB oxidation in alkaline media.

### 3.4. Rotating disk studies

**Figure 8:**
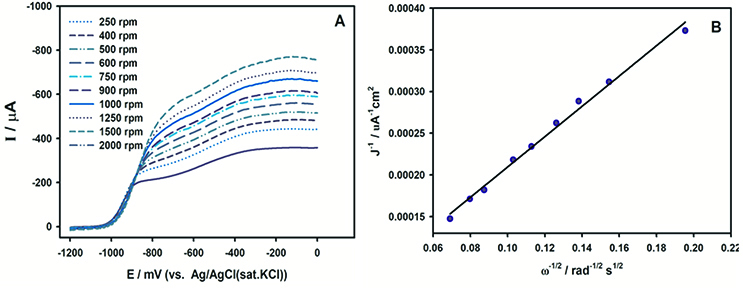
A) LSVs of rotating AgNPs/PAP/GCE in 2.0 M NaOH containing 5.0 mM DMAB solution at different rotation rates (250 to 2000 rpm). B) Koutecky–Levich (K-L) plot of AgNPs/PAP/GCE derived from Figure 8A.

The rotating disk electrode (RDE) technique is a convenient technique to determine the kinetics and the mechanism of electrochemical electrode reactions. Linear sweep voltammetric (LSV) studies were performed on AgNPs/PAP/GCE in the presence of DMAB to determine the kinetic parameters more quantitatively (Figure 8A). Based on the RDE results at a chosen potential, the number of electrons transferred in the redox reaction can be calculated using the Koutecky–Levich (K–L) equation (5) [65] as follows.

(5)1j=1jk+10.62nFACDMABDDMAB23ω12γ-16

where j is the measured current density, jk is the kinetic current density, ω is the electrode rotation rate (rad/s), n is the number of electrons transferred for DMAB, F is Faraday constant (96485) C mol ^-1^ ) , CDMAB is the concentration of DMAB in 2.0 M NaOH (5 ×10 ^-6^ mol cm ^-3^ ) , DDMAB is the diffusion coefficient of DMAB [13], γ is the kinematic viscosity of the solution (0.0117 cm ^2^ s ^-1^ ) which is determined with Oswald viscosimeter at 25 °C. According to the equations, the n was calculated from the slope of the Koutecky–Levich plot *j* ^-1^ vs. ω ^−1/2^ (Figure 8B). The calculated total electron number for DMAB oxidation is 5.6, which is close to the theoretical value of 6.

There are a limited number of studies on DMAB oxidation in fuel cell applications. These studies are as follows; the electrochemical behavior of DMAB on Au bulk electrode was investigated by Finkelstein et al. [18]. The results of this study showed that the total number of electrons transferred during the oxidation of DMAB was found as 5.4. This value depends on potential and formation of H2 bubble. The oxidation behavior of DMAB was studied by Rohan and Nagle using a gold microdisk in alkaline medium [13]. They exhibited that the electron number transferred in the electrooxidation reaction of DMAB varies from 3 to 6. It depends on the ratio of OH− to DMAB, with H2 evolution. An overall electron number of 6 have been determined with chronoamperometry results. Plana and Dryfe described the DMAB electrooxidation on polycrystalline gold electrodes at high pH [9]. They suggested two sequential three-electron processes on gold. At low potentials, three electrons are extracted from the molecule, denoting that hydrogen is not oxidized, whereas at high potential a six-electron oxidation occurs. In another study investigating the oxidation behavior of DMAB, Pt was used as the electrode [8]. Martins and Nunes evaluated the effect of DMAB concentration on the number of electrons transferred in the electrooxidation reaction. In the electrooxidation of DMAB on the Pt surface, the transferred electron numbers were calculated as 3.86, 4.68, and 3.76 from the discharge curves for three different DMAB concentrations. The reason for the low number of electrons is that the platinum surface catalyzes the formation of hydrogen, causing hydrolysis of DMAB. Sadik et al. explained electron transfer mechanism for the DMAB on gold surface in KOH solution [15]. This study has highlighted the pH-dependence of DMAB oxidation on gold in alkaline solution. The total number of electrons transferred in oxidation of DMAB on gold surface was calculated using the rotating disk electrode (RDE) as 5.74. All these studies examining the oxidation mechanism of DMAB show that the number of electrons transferred in electrode reaction on all gold-based surfaces is close to the theoretical number of 6. According to the results, it can be said that DMAB has direct oxidation reaction on gold surfaces. Conversely, when the platinum surface is used as the electrode, it is found that the number of electrons is reduced due to the formation of hydrogen. In our study, the number of electrons transferred in the DMAB oxidation on AgNPs/PAP/GCE surface was found to be 5.6. Although this value is consistent with the literature, it shows that direct oxidation reaction takes place. Furthermore, compared to gold and platinum electrodes, the proposed AgNPs-PAP composite electrode is advantageous in that it is relatively inexpensive, stable, and easy to prepare.

### 3.5. Stability of AgNPs/PAP/GCE

**Figure 9:**
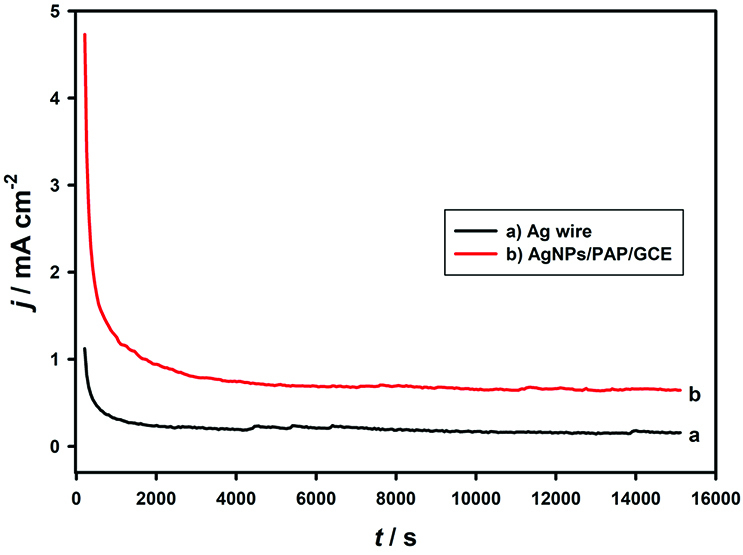
Stability of AgNPs/PAP/GCE and Ag wire in 2.0 M NaOH containing 8.0 mM DMAB for 15,000 s.

Stability is a challenging issue for fuel cell applications. For this purpose, chronoamperometry was applied at AgNPs/PAP/GCE and Ag wire to test the signal change during this measurement towards the response of 8 mM DMAB (Figure 9). In both chronoamperometric curves, the currents were dropped rapidly at first and then became relatively stable. The higher steady-state peak current was achieved at AgNPs/PAP/GCE as compared to the Ag wire. Moreover, a long-term storage of the AgNPs/PAP/GCE was investigated by keeping the electrode in vapor of the 2.0 M NaOH solution when no measurement was taken. The response showed no significant change in the peak current and peak potential of DMAB after 1 month. These results prove that AgNPs/PAP/GCE is a stable and efficient surface for the oxidation of DMAB.

## 4. Conclusion

In the present work, AgNPs/PAP/GCE catalyst has been prepared by electrochemical synthesis of AgNPs after electrochemical formation of poly aminophenol film on glassy carbon electrode surface. Morphological and chemical characterization of the AgNPs/PAP/GCE was carried out by SEM, TEM, EDX, XPS, and EIS techniques. The SEM images revealed that spherical AgNPs were distributed homogeneously on the PAP/GCE with the average size of approximately 100 nm. From the XPS results, it clarified that Ag species inside the PAP were chiefly dispersed as metallic Ag. Cyclic voltammograms of DMAB were indicated that higher electrocatalytic activity is obtained on the AgNPs/PAP/GCE as compared to the GCE and PAP/GCE in alkaline media. Experimental procedures such as cycle number of aminophenol polymerization, cycle number of Ag deposition, and NaOH concentration have also been optimized. The electrocatalytic oxidation of DMAB at AgNPs/PAP/GCE was found to proceed via 5.6 electron transfer, which was calculated from RDE results. It shows that direct oxidation reaction of DMAB takes place on AgNPs/PAP/GCE. The stability studies were performed by chronoamperometric technique and the results indicated that the AgNPs/PAP/GCE was introduced as a stable and efficient surface for the oxidation of DMAB. The overall results demonstrated that the AgNPs/PAP/GCE is a promising alternative as cheap, stable, and easy prepared anode material for direct DMAB fuel cells.
